# Data analysis for differentiating temporo mandibular disorders (TMD) pain from tooth pain

**DOI:** 10.6026/973206300200674

**Published:** 2024-06-30

**Authors:** Nishath Sayed Abdul

**Affiliations:** 1 Faculty of Oral Pathology, Department of OMFS and Diagnostic Sciences, College of Medicine and Dentistry, Riyadh Elm University, Riyadh, Kingdom of Saudi Arabia

**Keywords:** Temporomandibular disorders, endodontic therapy, TMD pain screener questionnaire

## Abstract

The factors differentiating temporomandibular disorders (TMD) pain from tooth pain is of interest to dentists. Prior to receiving therapy, participants answered the six-question TMD Pain Screener questionnaire. Applying validated Diagnostic Guidelines for TMD (DC/TMD), an orofacial pain specialist and endodontic resident with board certification performed endodontic and TMD examinations. In our study, sensitivity was higher for TMD screener regarding identification of all types of TMDs (0.94 (0.80-0.97), TMD pain not referring to teeth (0.94 (0.76-0.98), TMD pain referring to teeth only (0.94 (0.64-1.00)). TMD Pain Screener questionnaire can be applied in identification of TMDS in patient seeking endodontic therapy.

## Background:

Although it may seem similar to endodontic pain, temporomandibular disorders (TMD) prevalence and their role in endodontic patients is not being adequately studied [1, 2-3]. Toothache is the most common painful complaints in the orofacial area. Most toothache is attributed to odontogenic inflammation, within and around the tooth, and it constitutes one of the main causes of dental visits for patients[4, 5-6]. The word "odontogenic pain" refers to a variety of possible diagnoses, such as acute apical abscesses, painful apical periodontitis, and painful irreversible pulpitis being a pulpal diagnosis[3-5]. It is possible for a tooth pain to originate from a source other than the mouth due to pain being referred from other structures. TMDs have been found to transmit pain towards the dentoalveolar structures, for instance [6, 7- 8]. Patients with TMD may have pain, which may lead them to consult with different specialists. TMD is more prevalent among women because of hormonal fluctuations and the increased impact of psychosocial variables [7, 9, 10-11]. It is most commonly observed in individuals between the ages of 20 and 40years. Therefore, it can be said that TMD is an issue of civilization that may get worse because of a fast-paced lifestyle, constant stress, and improper operation of the masticatory apparatus [11, 12, 13- 14]. Stress is an undeniable contributing element, since it negatively impacts all masticatory structures and, if it persists, can reveal or exacerbate temporomandibular problems [15, 16, 17-18].

A study shows that there is diversity in the etiology of pain associated with TMJ disorders, including central and peripheral sources. Treatment choices should be guided by their discovery that TMD is associated with anatomical and functional alterations in the brain's prefrontal cortex, basal ganglia, and primary somatosensory cortex [19, 20- 21].Patients with TMD frequently experience their condition as a headache because of irregularities in TMJ, muscles of mastication, along with other nearby tissues [22, 23, 24-25]. The results of the research show that people who have tension headaches or migraines are more likely to experience typical TMD symptoms. Additionally, it has been demonstrated that individuals with TMD are more prone to get migraines, and that having both conditions at the same time makes each condition's symptoms worse [13-16]. It is difficult to diagnose and treat disorders of particular anatomical location. Patients frequently get referred to neurologists, otolaryngologists, dentists and surgeons [12-14]. There is little question that the identification and classification of illnesses may benefit from the engagement of numerous specialists in the issues impacting this field [15-18].

When a minimum of three of following indications are present, masticatory malfunction can be diagnosed: occlusal or non-occlusal parafunction, restricted mandibular mobility, discomfort and auditory sensations during mandibular motions, and difficulties opening the jaw [12-16]. The DC/TMD examination methodology should serve as the foundation for contemporary TMD diagnosis since accurate diagnosis is necessary for the development of an effective treatment plan [16-19]. A sizable fraction (12 to 50%) of patients seeking endodontic therapy at the dentist office does so due to NonoDontogenic or mixed origins of "tooth" pain [20-24]. The most frequent NonoDontogenic cause of "tooth" pain is temporomandibular joint (TMD) discomfort, which might originate from the masticatory muscle, adjacent tissues, or joints [12-15]. Dentists are skilled at identifying and treating odontogenic pain due to its prevalence. Dentists must take into account NonoDontogenic causes of pain when testing for dental pathology come back negative for an odontogenic complaint [15-18]. It may be useful to detect such individuals in routine dentistry practice by using a short, valid screening tool to help determine the most frequent NonoDontogenic cause of "tooth" pain in patients seeking endodontic therapy [18- 23]. There are some tools like TMD Pain Screener questionnaire and Dental Pain Questionnaire for identifying TMDS [19-25].But very few studies has been conducted to use these tools for identifying TMDS in patient seeking endodontic therapy. Therefore, it is of interest to evaluate the accuracy of a TMD Pain screener questionnaire in recognizing patient's TMD pain among patients seeking endodontic therapy for tooth pain.

## Methods and Materials:

We enlisted two hundred individuals who were seeking endodontic therapy for tooth pain. Prior to receiving therapy, participants answered the six-question TMD Pain Screener. Applying validated Diagnostic Guidelines for TMD (DC/TMD), an orofacial pain specialist and endodontic resident with board certification performed endodontic and TMD examinations.

## Inclusion criteria:

[1] Patients reporting to dental clinic with tooth pain

[2] Patient demanding endodontic therapy for complain of tooth pain.

## Exclusion criteria:

[1] Patients were not allowed in study if they

[2] Had a second diagnosis of concomitant orofacial pain;

[3] Possessed a medical history of severe orofacial injuries;

[4] Had a significant systemic disease, such as fibromyalgia, rheumatoid arthritis, or other widespread physical pain disorders, that was associated with altered pain sensitivity.

[5] Had prior experience with inter articular steroid injection or temporomandibular joint surgery

[6] We're not able to provide informed permission

## TMD screening questionnaire:

A self-report tool called the TMD screener was created to help individuals be screened for TMD that is associated to pain [19-25]. Using psychometric techniques for item selection, it was created in two versions: a long (6-item) version and a short (3-item) version. Its validity was assessed among 504 participants.

## Outcomes:

Sensitivity, specificity, positive predictive value (PPV), negative predictive value (NPV) were assessed on the basis of findings obtained and information gathered from the questionnaire filled by patients. Outcomes were analysed for three clinical situations:

[1] TMD pain not referred to teeth only

[2] TMD pain referred to teeth only

[3] All types of TMDS.

## Statistical analysis:

The data was tabulated and feed in MS excel sheet. The data was subjected for statistical analysis using SPSS version 21 software. The findings were presented in the form of confidence interval (CI) for parameters like Sensitivity, specificity, positive predictive value (PPV), negative predictive value (NPV).

## Results:

In our study, sensitivity was higher for TMD screener regarding identification of all types of TMDs (0.94 (0.80-0.97)), TMD pain not referring to teeth (0.94 (0.76-0.98)), TMD pain referring to teeth only (0.94 (0.64-1.00)). The specificity was slightly lower regarding identification of all types of TMDs (0.61 (0.42-0.78)), TMD pain not referring to teeth only (0.61(0.42-0.76)) and TMD pain referring to teeth only (0.72 (0.58-0.86)).The PPV for identification of all type of TMDs was0.72 (0.58-0.86), while it was0.64 (0.46-0.79) for TMD pain not referred to teeth. It was lower for TMD referring to teeth (0.46 (0.26-0.67)). The NPV values were 0.89 (0.68-0.96) for all types of TMDs pain, 0.92 (0.72-0.98) for TMD pain not referring to teeth only and 0.96 (0.78-1.00) for TMD pain referring to teeth only. The NPV values were lower than PPV values (Table 1, Table 2).

## Discussion:

The most frequent painful symptom in the orofacial region is toothache, accounting for 12% of cases. Toothache is one of the primary reasons individuals' visits the dentist and it is primarily caused by odontogenic inflammation inside and around the tooth [11-17]. Temporomandibular joint (TMD) pain is the most common NonoDontogenic cause of "tooth" pain, and it can come from the joints, surrounding tissues, or the masticatory muscle [13-18]. Odontogenic discomfort is common, thus dentists are trained to recognize and manage it. When testing for dental pathology yields negative results for an odontogenic complaint, dentists must consider NonoDontogenic causes of discomfort [12-16]. Using a brief, valid screening tool to identify the most common NonoDontogenic source of "tooth" pain in patients seeking endodontic therapy may assist identify such patients in routine dentistry practice[11-19]. The prevalence of TMD and their role in endodontic patients are not well studied, despite the fact that they may seem similar to endodontic pain [16-23]. To identify TMDS, there are tools such as the dental pain Questionnaire and the TMD Pain Screener questionnaire. However, there aren't many studies that employ these instruments to identify TMDS in patients undergoing endodontic therapy [24, 25]. This study was therefore conducted to evaluate the accuracy of a TMD Pain Screener questionnaire in recognizing patients TMD pain among patients seeking endodontic therapy for tooth pain.

A study found that tool like TMD Pain Screener questionnaire and Dental Pain Questionnaire showed significant sensitivity in diagnosing TMDS and differentiating it from endodontic pain [19]. Another study also showed similar results with TMD Pain Screener questionnaire [18- 24]. According to a study, the etiology of pain linked to TMJ issues is diverse and can come from both central and peripheral sources. The finding that TMD is linked to structural and functional changes in the brain's prefrontal cortex, basal ganglia, and primary somatosensory cortex should direct treatment decisions [21]. TMD patients often describe their condition as a headache due to abnormalities in the TMJ, the masticatory muscles, and other surrounding tissues [13-19]. The study's findings indicate that the likelihood of having typical TMD symptoms is higher among persons who suffer from migraines or tension headaches. Furthermore, it has been shown that those with TMD are more likely to experience migraines, and that the symptoms of both illnesses worsen when they coexist [14-18]. Difficulties pertaining to specific anatomical locations are challenging to diagnose and cure. Referrals to neurologists, otolaryngologists, dentists, and surgeons are common [12-18]. There is no doubt that the involvement of many professionals in the challenges affecting this subject may be beneficial for the identification and classification of disorders [13-19]. Masticatory dysfunction is diagnosed when at least three of the following symptoms are present: restricted mandibular mobility, occlusal or non-occlusal parafunction, discomfort and auditory sensations during mandibular motions, and difficulty opening the jaw [4-13]. Since an accurate diagnosis is essential to the creation of a successful treatment strategy, the DC/TMD evaluation approach ought to be the cornerstone of modern TMD diagnosis [5-8]. There is a significant portion of patients (12 to 50%) who come to the dentist for endodontic therapy because their "tooth" discomfort has NonoDontogenic or mixed etiology [12-19]. In our study PPV values was lower while NPV values were greater for TMD screener regarding identification of TMDs. The PPV for identification of all type of TMDs was 0.72 (0.58-0.86), while it was 0.64 (0.46-0.79) for TMD pain not referred to teeth. It was lower for TMD referring to teeth (0.46 (0.26-0.67)). The NPV values were 0.89 (0.68-0.96) for all types of TMDs pain, 0.92 (0.72-0.98) for TMD pain not referring to teeth only and 0.96 (0.78-1.00) for TMD pain referring to teeth only. The NPV values were lower than PPV values. Other studies also showed lower PPV values and higher NPV values for TMD screener [19]. Another study analysed TMD Pain Screener questionnaire and Dental Pain Questionnaire in patients with TMDs and tooth pain. They concluded that these two tools had lower PPV and higher NPV values. The findings are similar to findings of present study [20-]. The term "odontogenic pain" encompasses a range of potential diagnoses, including painful apical periodontitis, acute apical abscesses, and severe irreversible pulpitis, which is a pulpal diagnostic [11-14]. Because pain can refer from different structures, it is possible for a toothache to come from somewhere other than the mouth. For example, it has been observed that TMDs might transfer pain to the dentoalveolar tissues [15- 17]. TMD patients may have pain, which could prompt them to see various experts. Because of changes in hormones and the greater influence of psychosocial factors, TMD is more common in women [17- 23].The majority of cases are reported in people ages 20 to 40. Thus, TMD might be considered a product of civilization that could worsen due to a fast-paced lifestyle, on-going stress, and dysfunctional masticatory apparatus [10-15]. Given its detrimental effects on all masticatory structures and its potential to either disclose or worsen temporomandibular disorders, stress is an indisputable contributing factor [14-21]. These questionnaires continue to be used in epidemiological research intended to determine the magnitude of tooth pain frequency since the prevalence can be recalculated using the known error rates [11-15]. A word of concern should be issued because the huge confidence intervals (CIs) introduce a significant degree of uncertainty into the estimate, which may limit the applicability of this approach [10-18]. The best option for separating patients with odontogenic pain from those with TMD pain in clinical environments where TMD prevalence is not particularly low and for application in epidemiological studies is the 6-item TMD screener, which has a modest specificity and great sensitivity [3-7].

## Conclusion:

TMD Pain Screener questionnaire can be applied in identification of TMDS in patient seeking endodontic therapy.

## Figures and Tables

**Table 1:** Diagnostic Accuracy Measures of the Different Groups for the TMD Screener

	**All types of TMDS**	**TMDs not referring to teeth only**	**TMDS referring to teeth only**
Cell frequencies			
True positive	68	46	22
False negative	6	4	2
False positive	28	28	28
True negative	40	40	40
Prevalence of the target condition	0.54	0.42	0.27

**Table 2:** CI values of different Parameters for accuracy of TMD screener for three aims

	**All types of TMDs pain**	**TMD pain not referring to teeth only**	**TMD pain referring to teeth only**
Sensitivity (CI)	0.94 (0.80-0.97)	0.94 (0.76-0.98)	0.94 (0.64-1.00)
Specificity (CI)	0.61 (0.42-0.78)	0.61(0.42-0.76)	0.61 (0.42-0.76)
PPV (CI)	0.72 (0.58-0.86)	0.64 (0.46-0.79)	0.46 (0.26-0.67)
NPV (CI)	0.89 (0.68-0.96)	0.92 (0.72-0.98)	0.96 (0.78-1.00)
